# A randomized controlled trial on using predictive algorithm to adapt level of psychological care for community college students: STAND triaging and adapting to level of care study protocol

**DOI:** 10.1186/s13063-023-07441-7

**Published:** 2023-08-09

**Authors:** Alainna Wen, Kate Wolitzky-Taylor, Robert D. Gibbons, Michelle Craske

**Affiliations:** 1 Department of Psychiatry and Biobehavioral Sciences, University of California - Los Angeles, 760 Westwood Plaza, Suite 28-216, CA 90024 Los Angeles, USA; 2 Center for Health Statistics, University of Chicago, 5841 S. Maryland Avenue MC 2007, Office W260, Chicago, IL 60637 USA; 3 Department of Psychology, University of California - Los Angeles, 1285 Franz Hall, Box 951563, Los Angeles, CA USA

**Keywords:** Personalized treatment, Clinical trial, Digital phenotyping, Implementation, Depression, Anxiety

## Abstract

**Background:**

There is growing interest in using personalized mental health care to treat disorders like depression and anxiety to improve treatment engagement and efficacy. This randomized controlled trial will compare a traditional symptom severity decision-making algorithm to a novel multivariate decision-making algorithm for triage to and adaptation of mental health care. The stratified levels of care include a self-guided online wellness program, coach-guided online cognitive behavioral therapy, and clinician-delivered psychotherapy with or without pharmacotherapy. The novel multivariate algorithm will be comprised of baseline (for triage and adaptation) and time-varying variables (for adaptation) in four areas: social determinants of mental health, early adversity and life stressors, predisposing, enabling, and need influences on health service use, and comprehensive mental health status. The overarching goal is to evaluate whether the multivariate algorithm improves adherence to treatment, symptoms, and functioning above and beyond the symptom-based algorithm.

**Methods/design:**

This trial will recruit a total of 1000 participants over the course of 5 years in the greater Los Angeles Metropolitan Area. Participants will be recruited from a highly diverse sample of community college students. For the symptom severity approach, initial triaging to level of care will be based on symptom severity, whereas for the multivariate approach, the triaging will be based on a comprehensive set of baseline measures. After the initial triaging, level of care will be adapted throughout the duration of the treatment, utilizing either symptom severity or multivariate statistical approaches. Participants will complete computerized assessments and self-report questionnaires at baseline and up to 40 weeks. The multivariate decision-making algorithm will be updated annually to improve predictive outcomes.

**Discussion:**

Results will provide a comparison on the traditional symptom severity decision-making and the novel multivariate decision-making with respect to treatment adherence, symptom improvement, and functional recovery. Moreover, the developed multivariate decision-making algorithms may be used as a template in other community college settings. Ultimately, findings will inform the practice of level of care triage and adaptation in psychological treatments, as well as the use of personalized mental health care broadly.

**Trial registration:**

ClinicalTrials.gov NCT05591937, submitted August 2022, published October 2022.

**Supplementary Information:**

The online version contains supplementary material available at 10.1186/s13063-023-07441-7.

Depressive and anxiety disorders are highly prevalent, with high rates of non-recovery and recurrence [1]. Despite the existence of effective, evidence-based treatments, access to these treatments can be challenging, with long wait times and barriers such as cost and transportation [2, 3]. These challenges are particularly relevant for college students. A growing body of evidence suggests that depression and anxiety are prevalent and increasing among college students, particularly those of lower socioeconomic status [4]. Moreover, many college students face barriers to receiving mental health care [5].

One approach to improving psychological treatment access is stepped care models, where patients begin with low-intensity treatments (e.g., self-guided online bibliotherapy or online therapy supported by paraprofessionals^1^), and those who do not respond are moved to the next step of care (e.g., [6]). In stratified stepped care, patients with less severe symptoms are routed to low-intensity treatments, and those with more severe symptoms to high-intensity treatments (e.g., [7]). Meta-analyses of stepped care programs, most of which include stratification, yield modest effect sizes for depression [8, 9] and superiority over usual care for anxiety [10].

The Screening and Treatment for Anxiety and Depression (STAND) program was developed as a part of the Depression Grand Challenge (DGC) at the University of California - Los Angeles (UCLA) to address the need for accessible and efficient evidence-based psychological treatments. STAND provides evidence-based, stratified stepped care for depression and anxiety, ranging from a self-guided online wellness program, to coach-guided online cognitive behavioral therapy (CBT), to clinician-delivered psychological and psychiatric care [11, 12]. The current STAND program utilizes a traditional method of assigning and adjusting level of care based primarily on symptom severity. However, symptom severity alone, while useful, does not comprehensively capture all factors that predict treatment response or future need [13]. Moreover, any predictor variable in isolation will have limited predictive utility given that individuals can score positively on some predictors and negatively on others [14]. Therefore, the field has increasingly moved toward multivariate models that evaluate predictors in combination [15].

A plethora of factors can influence the level of care needed for an individual seeking psychological treatment. Evidence indicates the potential for (1) social determinants of mental health (e.g., race/ethnicity, housing security, discrimination, social support; [16], (2) early life adversity and life stress (e.g., childhood trauma, chronic stress; [17], (3) predisposing, enabling, and need influences on health service use (e.g., mental health stigma, beliefs about mental health treatment, [18], and (4) comprehensive mental health status (e.g., depression, anxiety, comorbidities, emotion dysregulation, [19]) to significantly impact engagement and response to treatment. A multivariate stratified stepped care approach that considers all of these factors may optimize the personalization of level of care triaging and adaptation. Moreover, these factors are all highly relevant to racial/ethnic minority groups, who experience greater barriers to psychological treatment [20]. Thus, there is a need for an examination of clinical care decision-making using a multivariate stratified stepped care approach that considers these factors comprehensively.

This randomized controlled trial (RCT), titled STAND Triaging and Adapting to Level of Care, aims to advance personalized mental health care for depressive and anxiety disorders, particularly for individuals of minority racial/ethnic backgrounds. This RCT is funded by the National Institute of Mental Health (NIMH) and is implemented through the ALACRITY Center, which focuses on optimizing the effectiveness, implementation, and sustainability of STAND. The primary objective of the Signature Project is to optimize the personalization of psychological treatments through a stepped care model based on multivariate stratification. Specifically, this RCT aims to compare the traditional symptom severity decision-making approach to a novel multivariate decision-making approach in mental health care. We propose the following aims:

- Aim 1: Triaging level of care. Evaluate whether a multivariate decision-making approach for triaging level of care using baseline measures of social determinants of mental health, early adversity and life stressors, influences upon health services use, and comprehensive mental health status, leads to greater treatment adherence and symptomatic and functional outcomes than a traditional symptom severity decision-making approach. We hypothesize that individuals in the multivariate condition will show greater adherence, greater improvements in depression and anxiety symptoms, and greater improvement in social, occupational, and academic functioning compared to the symptom severity approach. Moreover, we predict that between-group differences will increase annually with refinements to the multivariate algorithms.
- Aim 2: Adaptation algorithm development. Develop a predictive algorithm that combines individual baseline characteristics with time-varying indices of symptom severity, perceived support, life stressors, etc. to predict depression and anxiety symptom outcomes for adapting level of care (following initial triage). The multivariate algorithm will then be iteratively refined and tested for racial-ethnic and other modifiers.
- Aim 3: Adapting level of care. Evaluate whether the multivariate algorithm for adapting level of care based on time-varying indices lead to greater treatment adherence and outcomes than the traditional symptom severity adaptation. We hypothesize that compared to the symptom severity condition, the multivariate condition will show greater treatment adherence, greater improvements in depression and anxiety symptoms and in social, academic, and occupational functioning. We also predict that between-group differences will increase annually with refinements to the multivariate algorithm.

## Method

### Ethics approval, trial status, and protocol registration

This investigation has been approved by the University of California - Los Angeles Institutional Review Board (IRB #22–000205) and the Los Angeles County Department of Mental Health Human Subjects Research Committee (HSRC #365). This RCT started recruiting participants in August 2022. Recruitment will be conducted continuously over the course of 5 years, with an expected end date in 2027. The protocol was submitted for registration to ClinicalTrials.gov in August 2022 and was made available to the public on October 2022 (NCT 05591937). The ClinicalTrials.gov registration follows the guidelines for the World Health Organization Registration Data Set [21]. Substantial protocol amendments will be submitted to the Institutional Review Board for approval and updated on ClinicalTrials.gov. If amendments affect participants, they will be informed about the specific changes; if needed, additional consent will be requested. Non-substantial amendments will be recorded and filed using date identifiers, and if applicable, clinicians and staff will be notified of the relevant amendments.

### Participants

A total of *N* = 1000 participants, over five annual cohorts (*n* = 200 per year), will be recruited from a community college in the city of Los Angeles. We will include participants who are aged 18–40, have California state Medicaid insurance, have access to the internet and smartphone to access the virtual interventions and assessments, are able to comprehend the study materials, and agree to complete weekly assessments. We will exclude participants with diagnoses that require more specialized care (e.g., primary psychotic disorder, severe substance use disorder), marked cognitive impairment, severe neurological disorder, and current psychological and psychiatric treatment that the participant is unwilling to discontinue. Exclusion diagnoses will be assessed using the *Computerized Adaptive Testing for Substance Use Disorder* (CAT-SUD; [22], the *Prodromal Questionnaire – Brief Version* (PQ-B; [23], and sections of the *Structured Clinical Interview for the DSM* (SCID; [24]. Participants will complete online assessments through to 40 weeks in each annual cohort. Students will complete computerized tasks and self-report questionnaires at baseline and regularly throughout the duration of the study.

### Study design and timeline

**Fig. 1:**
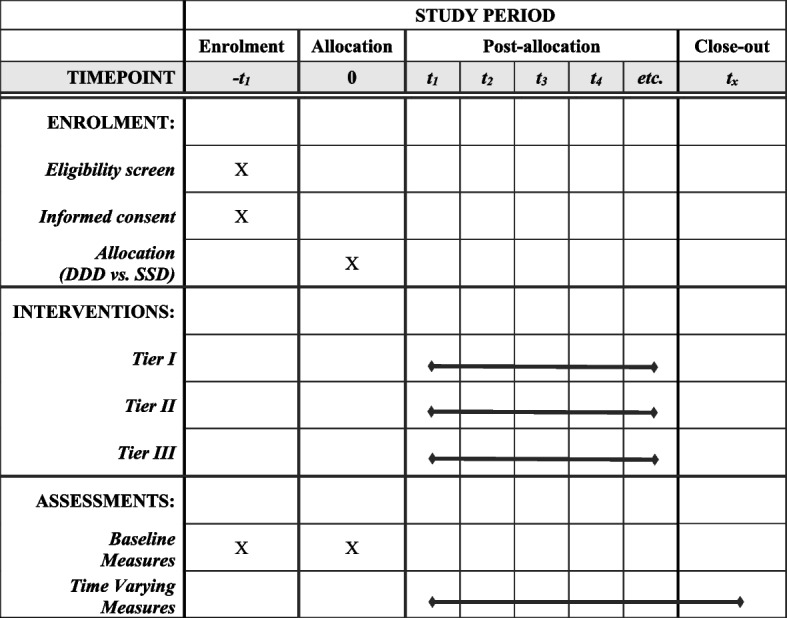
SPIRIT Flowchart of the Enrollment, Interventions, and Assessments. Note. See Table 1 for all baseline measures for level of care triaging algorithm. See Table 2 for all time-varying measures for level of care adaptation algorithm. Participants are followed up for 40 weeks post allocation. Tier I = self-guided online wellness program. Tier II = digital CBT with coaching. Tier III = clinician-delivered care. All tiers of treatment are offered both in the multivariate data-driven decision-making (DDD) condition and the symptom severity decision-making (SSD) condition to triaging and adapting level of care. SPIRIT = Standard Protocol Items: Recommendations for Interventional Trials

Participants will complete an online assessment to determine eligibility. Those who agree to participate, meet eligibility requirements, and enroll will be randomized to either the symptom severity or the multivariate condition for triaging and adapting level of care. Roughly 200 participants will be enrolled each year, for a total of 1000 participants over the course of 5 years. All participants in both conditions will receive the same treatments in STAND, which provides evidence-based, stratified stepped care for depression and anxiety, delivered virtually. The allocation across the treatment tiers in STAND will be as follows: 20 in Tier 1 online wellness program, 120 in Tier 2 digital therapy with coaching, and 60 in Tier 3 clinician-delivered care. This allocation is based on the distribution observed in the pilot trial at the same community college setting as the current investigation [11]. All participants will complete follow-up assessments through to 40 weeks (see Fig. 1).

In year 1, participants will be triaged to the initial level of care (self-guided online wellness program, digital therapy with coaching, or clinician-delivered care) according to either the symptom severity or the multivariate algorithm. Outcomes for the initial triaging phase will be assessed continuously for 8 weeks, with the main outcome assessment on between-group differences at week 8. In year 1, the decision to adapt level of care (following initial triage) will align with typical clinical models that wait for a course of treatment to “fail” before making adaptation decisions [6, 25], and thus will be made at the 16-week assessment for all participants and based on symptom severity. Data gathered from year 1 will be used to generate the predictive algorithms for adaptation to the level of care to be used in subsequent years for those assigned to the multivariate condition.

Hence, from year 2 through year 5, participants in the multivariate decision-making condition will not only be triaged to the initial level of care using multivariate models but will also receive multivariate adaptation every 4 weeks starting at week 8. Participants in the symptom severity decision-making condition will continue to be considered for adaptation based on symptom severity alone at week 16. Outcome for the adaptation phase will be assessed from week 16 to week 40. Data accrued will be used to update and refine the algorithms annually to optimize their predictive accuracy.

### Randomization and treatment tier assignment

Each year, 200 participants will be randomized into the symptom severity decision-making (SSD) condition or the multivariate, data-driven decision-making (DDD) condition until each tier reaches capacity. This includes the Tier 1 online wellness program (capacity: 10 SSD vs. 10 DDD), Tier 2 digital therapy with coaching (capacity: 60 SSD vs. 60 DDD), and Tier 3 clinician-delivered care (capacity: 30 SSD vs. 30 DDD). These capacities were selected to reflect the natural distribution during the pilot trial that took place in the same community college setting as the proposed study [11].

Simple randomization to either the DDD or the SSD will be conducted using a python randomization function [26] by the research team programmer. Allocation concealment will be ensured, as the randomization function will not be run until the participant completes baseline assessments. Thus, randomization will be conducted without influence of the principal investigators, research staff, or clinicians. The design of the RCT allows the conditions (DDD vs. SSD) to be double blinded. Specifically, because three tiers of treatment are available in both the DDD and the SSD, the conditions will be feasibly masked to the participants as well as the research and clinical staff. For instance, the statistician will be able to access the condition variable, coded as 0 and 1, but will be blinded to what each condition represents. Because the blinding and masking pertain to the approach used for tier assignment and not the treatments themselves, no emergency unblinding procedure is needed.

### Algorithm development procedures

#### Algorithm development for multivariate data-driving decision-making (DDD)

Aim 2 of the current investigation centers on the development and refinement of the multivariate decision-making algorithm for initial triage and treatment adaptation. Specifically, the triaging algorithm will be refined based on the algorithm developed in the pilot trial [11], and the adaptation algorithm will be developed for the current investigation. The DDD algorithm for triage will consider the static baseline features, including social determinants of mental health, early adversity and life stress, predisposing, enabling, and need influences on health care service use, and comprehensive mental health status, as detailed below. To maximize the predictive accuracy between baseline characteristics and week 8 symptoms, we will explore models that bridge the gap between traditional statistical models and machine learning models (e.g., regularized regression models) that will allow us to identify a smaller set of baseline predictors that preserve the predictive accuracy of the total set of predictors. Model selection will be based on minimizing the root mean square error (RMSE) between the observed outcomes and the estimated outcomes at week 8. For count variables such as treatment adherence, we will use regularization methods for generalized linear models.

The DDD algorithm for adaptation will consider the static baseline features as well as the current level of care and time-varying predictors (e.g., symptom severity, life stress, perceived support, treatment adherence) over 4-week epochs to predict outcomes 4 weeks later (e.g., weeks 1–4 predict week 8 outcomes, weeks 1–8 predict week 12 outcomes). We will first fit generalized linear mixed-effect regression models to derive empirical Bayes estimated 8-week trajectories based on the initial 4 weeks of data. To predict future outcomes from baseline data, current tier, and weeks 1–12 outcomes, we will use the model estimates for the fixed-effects (baseline and current tier) and the Bayes estimates [27] for the random effects that reflect the outcome trajectory assessed through 12 weeks. These fixed and random coefficients will then be used to predict the distal endpoint (16 weeks) and be used to perform treatment adaptation. Once calibrated, we will use this predictive model to set thresholds on the level of change that will lead to adaptation of level of care (e.g., no change, re-engage in care, increase level of care) at 4-week intervals of treatment. We will update and refine the algorithm annually over the 5 years to optimize its predictive accuracy and utility as a decision-making tool. The final model will be derived using model selection criteria designed to identify the most useful subset of variables that maximizes predictive accuracy.

#### Utilization of symptom severity decision-making (SSD)

The SSD for triage and adaptation will consider only the symptom severity at baseline and week 16, respectively. Specifically, triaging will be determined by scores on the *Computerized Adaptive Tests for Major Depressive Disorder* (CAT-MDD; [28], *Anxiety* (CAT-ANX; [29], and *Suicide Scale* (CAT-SS; [30]. The CAT-MDD and CAT-ANX will be used to provide cutoffs for none, mild, moderate, and severe levels of symptoms. The CAT-SS will identify participants who indicate suicidal ideation and either intention or plan. Participants with less than mild depressive or anxiety symptoms and no suicidality risk will be triaged to Tier 1 for the self-guided online wellness program; those with mild to moderate depressive symptoms or mild to severe anxiety symptoms and no suicidality will be triaged to Tier 2 for digital CBT with coaching; participants with severe depressive symptoms or suicidality will be triaged to Tier 3 for clinician-delivered care. At week 16, scores on the CAT scales will be used to determine if participants should move to a higher level of care.

### Variables used in the multivariate predictive algorithm

Predictor measures for both triage and adaptation algorithms will be assessed online. All measures will be assessed at baseline, and some will be additionally measured either weekly or every 8 weeks. See Table 1 for baseline static measures that will be used in the predictive algorithm for triaging to level of care, and Table 2 for time-varying measures that will be used in the algorithm for adapting level of care. Wherever possible, the measures utilized are adapted from the Healthy Minds Survey, a large-scale mental health assessment that has been implemented in community colleges since 2016 [31].

**Table 1:** Baseline measures for the level of care triaging algorithm

Construct	Measures
Age, sex at birth, gender identity, race/ethnicity, sexual orientation, citizenship/immigration status	Healthy Minds Survey (1 item each)
Family pride	^a^Family Environment Scale – Family Pride/Familism Subscale (2 items)
Family cultural conflict	^a^Hispanic Stress Inventory – Family Cultural Conflict Subscale (2 items)
Discrimination	^a^Major Experiences of Discrimination-Abbreviated scale (12 items)
Employment	Healthy Minds Survey (1 item)
Housing security, food security, financial stress	Healthy Minds Survey (1 item each)
Social support	Medical Outcomes Social Support Scale (4 items)
Early adversity and life stress	Adverse Childhood Experiences Questionnaire (10 items); Youth Partners in Care Life Events Scale (18 items)
Beliefs about mental health treatment and stigma	Healthy Minds Survey (4 items)
Willingness to pay for mental health treatment, insurance status	Healthy Minds Survey (1 item each)
Perceived need for mental health treatment	Healthy Minds Survey (1 item)
Depressive symptoms	Computerized Adaptive Tests – Major Depressive Disorder
Anxiety symptoms	Computerized Adaptive Tests – Anxiety
Suicidality and self-harm	Computerized Adaptive Tests – Suicide Scale
Sleep	Insomnia Severity Index (7 items)
Substance use	Computerized Adaptive Tests – Substance Use
Other medical and mental health comorbidity and chronicity	Screening Assessment for Guiding Evaluation-Self Report; Healthy Minds Survey (1 item)
Emotion dysregulation and regulatory strategy use	^a^Difficulties in Emotion Regulation Scale (5 items); ^a^Cognitive Emotional Regulation Questionnaire (9 items)
Neurocognitive functioning	Test My Brain
Mental health treatment history and preferences	Healthy Minds Survey (5 items)
Social, occupational, and academic functioning	^a^Work and Social Adjustment Scale (2 items); Healthy Minds Survey (2 items)

^a^ = scales were abbreviated versions of the original measures adapted for the current investigation

**Table 2:** Time-varying measures for the level of care adaptation algorithm

Variable	Measure	Frequency
Symptom severity for depression, anxiety, suicidality	CAT-MDD, CAT-ANX, CAT-SS	Weekly
Symptom severity for substance use	CAT-SUD	Weekly
Current life stress	1-item perceived stress rating	Weekly
Food/housing security and financial stress	Healthy Minds Survey	Weekly
Perceived support	Medical Outcomes Social Support Survey	Weekly
Treatment adherence	# sessions/lessons completed	Weekly
Dimensional measures for depressive and anxiety symptoms	Patient Health Questionnaire; Generalized Anxiety Disorder Scale; Fear Questionnaire – Agoraphobia Subscale; Social Anxiety Disorder Dimensional Scale; Panic Disorder Severity Scale	Every 2 weeks
Functioning level	^a^ Work and Social Adjustment Scale	Every 8 weeks
Employment	Healthy Minds Survey	Every 8 weeks
Discrimination	^a^ Everyday Discrimination – Short Form	Every 8 weeks
Family cultural conflict	^a^ Hispanic Stress Inventory – Family Cultural Conflict Subscale	Every 8 weeks
Sleep	Insomnia Severity Index	Every 8 weeks
Emotion regulation	^a^ Cognitive Emotion Regulation Questionnaire	Every 8 weeks

*CAT* computerized adaptive testing, *MDD* major depressive disorder, *ANX* anxiety, *SS* Suicide Scale, *SUD* substance use disorder

^a^ = scales were abbreviated versions of the original measures adapted for the current investigation

#### Social determinants of mental health

Social determinants of mental health have been linked to psychopathology, treatment seeking, and treatment engagement/response [32, 33]. Baseline static variables that will be assessed include age, sex assigned at birth, gender identity, sexual orientation, race/ethnicity, and citizenship/immigration status. Family pride at baseline will be assessed using two items adapted from the* Family Environment Scale – Family Pride/Familism Subscale* in the National Latino and Asian American Study; items are scored from 1 to 4, with higher scores reflecting greater pride (7 items, [34]. Experiences of discrimination due to racial, ethnic, socioeconomic, gender, or other reasons will be assessed at baseline using the *Major Experiences of Discrimination – Abbreviated scale* (12 items); items are scored from 1 to 4, where higher scores reflect more frequent experiences of discrimination [35]. Family cultural conflict will be assessed at baseline and every 8 weeks using two items based on the *Hispanic Stress Inventory – Family Cultural Conflict Subscale*; items are scored from 1 to 3, with higher scores indicating higher levels of conflict [36]. These items were validated in both US-born individuals and immigrant individuals [36, 37]. Measures that will be assessed every 8 weeks include employment, financial stress, food security, and housing security (one item each). Questions assessing food and housing security and financial stress are scored dichotomously (1 = *yes* and 0 = *no*). Experiences of discrimination will be assessed every 8 weeks using three items adapted from the *Everyday Discrimination Scale – Short Form* (6 items); items are scored from 1 to 6, with higher scores reflecting more discrimination experiences [38]. Perceived social support will be measured weekly using the *Medical Outcomes Social Support Scale* (4 items); items are scored from 1 to 4, where higher scores reflect higher support level [39].

#### Early adversity and life stress

Both early adversity and ongoing life stress are strongly related to mental health [17, 40]. At baseline, adverse experiences will be measured using the *Early and Recent Adversity Questionnaire* (40 items); items are rated with *yes* = 1 and *no* = 0 responses, with a specification on the number of times an adverse experience has occurred for items that are endorsed [41]. Life stress in the past 6 months will be measured using the *Youth Partners in Care Life Events Scale* (18 items); items are rated with *yes* = 1 and *no* = 0 responses [42]. Ongoing life stress will be measured weekly using a single item on perceived stress; the item is scored from 0 to 3, where higher scores reflect greater perceived stress. The single item has shown convergent validity with more comprehensive stress measures [43].

#### Predisposing, enabling, and need influences on health care service use

The measures used to assess factors that influence health care service use are guided by the Andersen Behavioral Model of Influences upon Health Service Use [44], with many constructs overlapping with the social determinants of mental health and with early adversity and life stress. Non-overlapping measures that will be assessed separately include beliefs about and attitudes toward mental health treatment (2 items) and perceived and personal stigma about receiving mental health treatment (2 items). These items were adapted from the *Perceived Devaluation – Discrimination scale* [45, 46]). Enabling factors of pricing and cost of services will be measured with one item on insurance status and one item on willingness to pay for mental health services. Perceived need for mental health service will be assessed using one item.

#### Comprehensive mental health status

Baseline variables include mental health comorbidity and chronicity, which will be assessed using the *Screening Assessment for Guiding Evaluation – Self-Report* (SAGE-SR; [47]. Physical health will also be assessed at baseline using one item. Emotion dysregulation, which is associated with many forms of psychopathology, will be assessed using five items adapted from the *Difficulties in Emotion Regulation Scale—Short Form* (DERS; 16 items); scores range from 1 = *Almost Never* to 5 = *Almost Always*, where higher scores reflect greater regulatory difficulty [48, 49]. The use of specific regulatory strategies will be assessed every 8 weeks using a brief version of the *Cognitive and Emotion Regulation Questionnaire* (CERQ; 9 items) developed for the current study; scores range from 1 = *Almost Never*, to 5 = *Almost Always*, where higher scores reflect more frequent regulatory strategy use [50]. Both the DERS and the CERQ have demonstrated adequate psychometric properties [51, 52]. Neurocognitive functioning will be assessed at baseline via *TestMyBrain.org*, an online assessment platform that has subtests on *Vocabulary, Matrix Reasoning, Choice Reaction Time, Graduate Onset Continuous Performance, Digit Symbol Matching,* and *Multiple Object Tracking* [53]. The *Multiracial Emotion Identification* test will also be administered to assess emotion recognition and social perception. All of the subtests have high reliability [54]. Treatment history and preference will be assessed at baseline using five items, including an item on preference for online vs. clinician-delivered therapy. Lastly, because abnormalities in sleep–wake behaviors are common in mood and anxiety disorders, sleep will be assessed every 8 weeks using the *Insomnia Severity Index* (ISI; 7 items); items are scored from 1 to 5, where higher scores reflect poorer sleep quality [55]. The ISI has demonstrated excellent psychometric properties [56].

### STAND treatment

Participants in both the SSD and DDD conditions for triaging and adapting level of care will receive the same treatment in STAND. Interventions comprise of three levels or tiers, all of which are delivered virtually.

#### Tier I—Self-guided online wellness program

The lowest tier of treatment is an online wellness program consisting of self-guided online CBT prevention strategies. This online program was adapted from a web-based rumination-focused CBT program, which has demonstrated efficacy for depression and anxiety in college samples [57, 58]. In the current study, the online wellness program focuses on reducing perseverative thinking, learning coping skills, and building resilience.

#### Tier II—Digital CBT with coaching

**Fig. 2:**
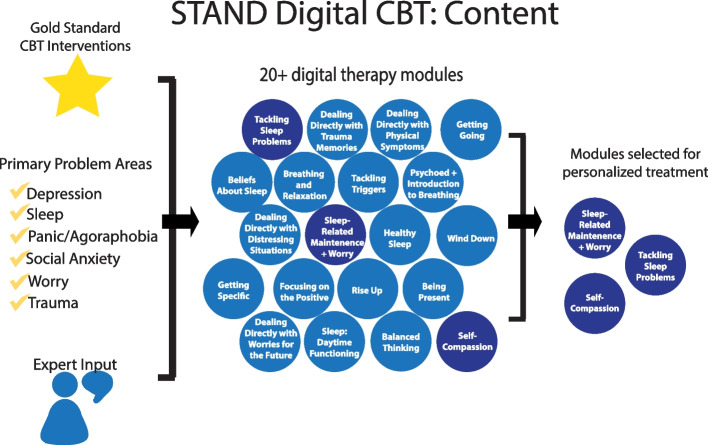
Online cognitive behavioral therapy modules in STAND

The middle tier consists of online CBT modules developed by the UCLA DGC [12], supported by coaches through video chats. All modules are evidence-based and are formatted into a unified approach for depression, anxiety and worry, panic, social anxiety, trauma, and sleep dysregulation. Digital CBT has established effectiveness for depression, anxiety, and stress in college students [59]. In STAND, the embedded measurement-based systems direct participants to the treatment content that are most relevant to their concerns (see Fig. 2). Specifically, treatment content will be recommended based on dimensional symptom measures that will be completed every 2 weeks. Specifically, depressive symptoms will be assessed using the *Patient Health Questionnaire* (PHQ-9; [60], anxiety symptoms will be assessed using the *Generalized Anxiety Disorder Scale* (GAD-7; [61], agoraphobia symptoms will be assessed using the *Fear Questionnaire – Agoraphobia Subscale* (FQ-AG; [62]); social anxiety symptoms will be assessed using the *Social Anxiety Disorder Dimensional Scale* (SAD-D; [63], and panic symptoms will be assessed using the *Panic Disorder Severity Scale* (PDSS; [64].

CBT skills in Tier II include behavioral activation, exposure therapy, cognitive restructuring, self-compassion, relaxation, mindfulness, and sleep regulation. Participants complete a 30–40-min online lesson that teaches skills through text, graphics, audio, video, and quiz content, designed to facilitate retention. Coaches will be recruited from the same community college, and they will provide up to eight 30-min one-on-one video sessions to their assigned participants. Coaching improves the retention and outcome from digital mental health treatments [65]. Specifically, coaches will use core process skills and motivational interviewing to increase engagement, support and encourage the application online lessons or CBT skills, and problem solve barriers to skill utilization. The coaches will be trained and certified over 15 weeks in foundational CBT skills, active listening, empathic responding, motivational support, confidentiality, and ethical decision-making. During the training phase, coaches are required to engage in 5–10 h per week of instruction and assignments and to attend weekly group lessons with roleplay practice, and evaluative feedback. To receive certification, coaches must demonstrate above a threshold of competency in core process skills and module integration through a mock coaching demonstration. All coaches receive weekly supervision from a licensed clinician. Homework exercises for participants completing the digital therapy are supported by an app toolbox accessible on smartphones and mobile devices. Spanish translation will also be available to enhance the accessibility of the materials, given that there is a large Spanish-speaking population at the community college wherein recruitment will occur.

#### Tier III—Clinician-delivered care

The highest tier provides evidence-based clinician-delivered CBT modules (see Table 3). Therapy is individualized, using a principle-based approach to treatment (as opposed to a manualized, multi-component “recipe” for treatment). The specific psychotherapy modules utilized are chosen based on a process-based functional assessment that identifies a principal target (e.g., inactivity/low mood) and a corresponding first-line treatment (e.g., behavioral activation) that maps onto that target. Modules are selected one at a time (i.e., no multi-component treatment packages are used). Similar to Tier II, treatment module selection in Tier III will consider dimensional symptom measures that will be completed every 2 weeks. This includes the same assessments of depressive symptoms (PHQ-9; [60], anxiety symptoms (GAD-7, [61], agoraphobia symptoms (FQ-AG, [62]), social anxiety symptoms (SAD-D; [63], and panic symptoms (PDSS, [64]. If symptoms do not improve with a first-line treatment module, a second-line treatment module will be considered for the same target process (e.g., worry). If an updated functional assessment suggests a different principal process to target (e.g., from targeting worry to targeting trauma-related symptoms), treatment module will also be selected and adjusted accordingly. Therapy can be complemented by protocolized medication management as needed. Sessions are delivered via telehealth. To begin providing therapeutic treatment, all clinicians must demonstrate adequate competency in the CBT modules. All clinicians will receive weekly supervision from a licensed clinician. Specifically, clinicians will engage in case conceptualization and treatment planning with a licensed clinician prior to starting psychotherapy with each participant.

**Table 3:** Clinician-delivered treatment in STAND

Problem area	Process targeted	First-line therapy module + medication as appropriate	Process being targeted	Second-line therapy module + medication as appropriate
**Low Activity/Sadness**	Low response contingent positive reinforcement	Behavioral activation (mood monitor, activity schedule, problem solve barriers, sleep schedule for barriers)	Cognitive distortions; rumination	Cognitive restructuring; OR mindfulness, value-driven action; OR problem solving
**Anhedonia**	Reward hyposensitivity	Pleasant event scheduling (hedonic and eudaimonic rewards), memory specificity recounting	Reward hyposensitivity	Cognitive restructuring with positive focus; cultivating positivity
**Fear/Phobia**	Deficits in extinction safety learning; avoidance	Exposure therapy	Negative cognitive bias; poor social skills	Cognitive restructuring; OR mindfulness, value-driven action; OR social skills training
**Worry**	Negative cognitive bias	Cognitive restructuring OR mindfulness, value-driven action	Avoidance (experiential, in vivo)	Exposure therapy; OR mindfulness, value-driven action; OR social skills training
**Sleep Dysregulation**	Sleep dysregulation	Brief behavioral therapy for insomnia	Negative cognitive bias	Cognitive restructuring
**Trauma—Fear**	Deficits in extinction safety learning; avoidance	Imaginal and in vivo exposure	Negative cognitive bias	Cognitive restructuring and impact statement
**Trauma—Guilt, Shame, Cognitive Distortions**	Negative cognitive bias	Trauma narrative with cognitive restructuring and impact statement		
**Chronic Suicidality, Self-Harm, Affective Instability**	Low tolerance of distress	Distress tolerance skills	Poor emotion regulation; interpersonal difficulties	Emotion regulation skills; interpersonal effectiveness skills
**Mania**	Circadian dysregulation	Brief behavioral therapy for insomnia		
**Major Life Stressors (any symptom profile)**	Poor coping	Problem solving for controllable stressor; mindfulness, value-driven action for uncontrollable stressor		
**Interpersonal Relations (any symptom profile)**	Social skills deficits	Social skills training		

Medications are included in the treatment as appropriate

#### Treatment discontinuation or modification

There are several conditions in which enrolled participants will be removed from the STAND treatment. This includes participants who show rapid symptom deterioration or new psychotic or severe substance use disorder symptoms and require a higher level of care than STAND provides (e.g., partial hospitalization program, residential care). Participants who choose to initiate or re-initiate care with another mental health provider may also be removed from the study. Lastly, participants who consistently do not respond to repeated suicide risk management calls may have their clinical care transferred or discontinued as appropriate, but they will be able to remain in the study to complete CAT-MH and other web-based assessments should they be interested.

#### Treatment integrity and fidelity

Individualized training and support will be offered for coaches who provide the tier 2 digital mental health intervention and clinicians who provide the tier 3 clinician-delivered intervention. Coaches and clinicians will receive standardized training on intervention delivery and ongoing supervision to maximize adherence to protocols. Specifically, tier 2 coaches (15 to 40) will receive extensive training via didactics and roleplay practice focused on CBT module content as well as training on empathetic responding, active listening, ethical decision-making, and motivational support. Similarly, tier 3 clinicians (6 to 9) will complete extensive training in the form of didactics and roleplay practice on therapy module selection and delivery as well as medication management. Trainers for coaches and clinicians will assign scores based on level of competence for each digital lesson/therapeutic module, and a threshold must be met for certification. After completing the training, coaches and clinicians will meet with their respective supervisors weekly for consultation and training or skills practice as needed. Supervision for coaches will be provided by licensed social workers who have been training coaches in the pilot trial at the same community college [11]. Supervision for clinicians will be provided by a psychologist and a psychiatrist who have undergone STAND training.

Several measures will be implemented to monitor and increase fidelity for coach-delivered digital mental health sessions and clinician-delivered psychotherapy. Coaches will use a checklist to guide the content of their coaching sessions and rate their performance after each coaching session. Supervisors will also review a portion of the coach’s sessions and rate their performance using a standardized form that parallels the self-ratings. Feedback from these ratings will be shared with the coaches to improve adherence and competency. Similarly, clinicians will record their modular approaches for each participant. Trained clinical research staff will review a portion of the clinician’s sessions, rate their performance using a standardized form, and provide feedback to the clinicians. Participants will also be asked to complete surveys where they will report the topics covered by their clinicians, which will be used to assess the fidelity and face validity of the clinician-delivered care.

### Outcome measures

We will examine the following multidimensional outcome measures: adherence to treatment, symptoms of depression and anxiety, and social, occupational, and academic functioning. These measures will be assessed at baseline and through to 40 weeks. Data up to week 8 will be used for analyzing the outcomes from DDD vs. SSD for the initial triage to level of care, and data up to week 16, week 24, week 32, and week 40 will be used for analyzing the outcomes from DDD vs. SSD for the later adaptation to level of care.

#### Adherence

The primary operationalization for adherence will be the total number of clinician sessions, coaching sessions, or online lessons completed. The total number of missed/canceled sessions with clinicians or coaches, the total number of times logged on, and the total time spent in the online lessons will be secondary measures. Measures of adherence will be reported weekly.

#### Symptoms

Depressive and anxiety symptoms will be measured weekly using the CAT-MDD and CAT-ANX [28, 29]. Suicidality will be assessed weekly using the CAT-SS [30]. A total score for depression, anxiety, and suicidality will be generated separately by the adaptive tests, ranging from 0 to 100. In general, CAT scales have demonstrated excellent convergent validity with structured clinical diagnostic interviews and gold-standard symptom measures [66]. The CAT-MDD and CAT-ANX have been validated against hour-long SCID diagnostic interviews for DSM-5; the CAT-SS has been validated against structured clinical interviews and shown to predict future suicide attempts with high accuracy [67–69].

#### Functioning

Two items will be used to assess functioning at work/school and in social relationships. Items were based on the *Work and Social Adjustment Scale* (5 items; [70]. A sum score will be computed, reflecting general functioning. The scale has excellent internal consistency, test–retest reliability, and convergent validity and is sensitive to treatment [71]. Grade point average and perceived impact of mental health on academic functioning will be measured with one item each, developed by the current investigation. Functioning measures will be completed by participants every 8 weeks.

### Data analysis

Analytical approaches will focus on the aims of the current investigation, which is to develop and refine predictive algorithms for triaging and adapting level of care using the novel multivariate approach and compare it to the traditional symptom severity approach. Analyses will be done separately for triaging to the initial level of care and adapting the level of care. In both the SSD and DDD, baseline measures (symptoms only for SSD, comprehensive set of variables for DDD) will be used in the statistical models that guide triaging to the initial level of care. The treatment response to the initial triaging will be based on outcome measures assessed at week 8, where we will also compare the SSD and DDD with respect to outcomes. Statistical models that guide adapting level off care will be conducted at week 8 and will occur every 4 weeks until the end of the trial at week 40 for the DDD, and at week 16 for the SSD. Adapting level of care in DDD will be based on baseline characteristics, current tier level, and additional dynamic variables collected over 4-week epochs. Adapting the level of care in SSD will be based on current symptoms and current tier level only. The treatment response to the adaptation will be based on data collected 4 weeks later, where the SSD and DDD approaches will be compared.

#### Analytical approach for aim 1

The focus of Aim 1 is to compare the multivariate approach for triaging to initial level of care with the symptom severity approach with respect to treatment outcomes. Regarding algorithm refinement for the multivariate approach, we will explore analytical approaches that bridge the gap between traditional statistical models and machine learning models to identify a subset of predictors that accurately predict week 8 outcomes based on baseline measures. This would involve using regularized regression models—including ridge regression, lasso regression, and elastic net regression—that allow for the identification of a smaller and more manageable set of baseline predictors that preserve the predictive accuracy of the full set of predictors [72–74]. Model selection among models with varying sets of predictors will be based on minimizing the root mean square error (RMSE) between the observed outcomes at week 8 and the estimated outcomes. Regularization methods for generalized linear models will be used for count variables [75, 76].

At the end of each academic year, variables included in the predictive model will be reviewed, and variables will be removed as appropriate. These changes will be reflected in the next academic year at the community college, starting in August. The annual review will also allow us to identify additional predictors for the outcomes that will be added as new data become available.

Outcome analysis will be conducted to compare the DDD vs. SSD with respect to symptom severity, functioning level, and treatment adherence at week 8. Linear regression models will be used to compare the two conditions on symptom severity and functioning level, whereas generalized linear models (Poisson and binomial regression models) will be used for treatment adherence. We will examine the main effect of year and the condition (SSD, DDD) × year interaction, to determine if the between-group effects are increasing over time with increased experience and algorithmic improvements. We will conduct the outcome analyses in three ways that differ in the treatment of participants with off-protocol shifts (e.g., receive treatments outside STAND, discontinued STAND clinical care). The “intent-to-treat” analysis will include all participants; the “per protocol” analysis will remove participants with off-protocol shifts; the “as-treated analysis” will include all participants, but off-protocol shift will be accounted for as a covariate variable (*yes* = 1, *no* = 0).

Participants who require a higher level of care due to safety concerns when their condition does not allow it at the time will be considered “treatment failures.” This is because the participant was unable to safely remain in their assigned level of care due to symptom severity or worsening. Their treatment failure is an important outcome for consideration. Thus, a secondary analysis will be conducted using mixed-effects logistic regression analysis to compare SSD and DDD with respect to rate of treatment failures over time. We will also model time to first treatment failure using a Cox regression model. As another secondary analysis, we will explore whether treatment adherence mediates symptom outcomes. A linear mixed model will be conducted with treatment adherence included as a time-varying covariate. We will then examine whether this mediated effect is moderated by treatment tier, as adherence may be more relevant to outcomes for individuals allocated to higher than lower tiers of care.

#### Analytical approach for aim 2

The focus of Aim 2 is to develop a multivariate predictive algorithm to be used for adapting level of care. We will use linear mixed-effects regression models to develop the predictive algorithm. The model will include baseline measures, current tier, and time-varying measures over accumulating 4-week epochs as predictors; symptoms, functioning, and treatment adherence 4 weeks later will be included as outcomes. For example, the prediction of week 8 outcomes will include baseline variables, current tier, and time-varying measures from weeks 1 to 4. We will use model-based estimates for fixed-effects and Bayes estimates for random effects to describe the trajectory of the outcome based on the repeated assessments [27]. These estimates will then be used to estimate the outcome at the distal endpoint (e.g., week 8). Treatment adaptation will be conducted based on this estimated outcome. Model selection for treatment adaptation will be based on minimizing the RMSE between the observed outcomes and the estimated outcomes. We will then use this predictive model to set thresholds on what level of change or lack thereof will lead to adaptation of level of care (i.e., tier shift) at 4-week intervals. Sensitivity analysis will be performed in the generalized linear mixed models to determine if there is significant variance associated with the clustering of students within coaches/clinicians.

The predictive algorithm will be designed to predict the lowest level of care that would produce improvement in symptoms at the outcome point. For example, if the predicted outcome at week 8 for a Tier I participant suggests that the participant will show greater improvements in symptoms if they were assigned to either Tier II or Tier III, then Tier II will be recommended. In contrast, if a Tier I participant’s predicted outcome may not be improved in Tier II but would be improved in Tier III treatment, then Tier III would be recommended.

#### Analytical approach for aim 3

Like Aim 1, outcome analyses for adapting level of care will be conducted in three ways. This includes intent-to-treat analysis (all participants are included regardless of off-protocol shifts), per protocol analysis (participants with off-protocol shifts are excluded from the analysis but retained in the algorithm), and as-treated analysis (inclusion of off-protocol shift as a covariate). We will statistically compare outcome trajectories through 5 years between the SSD and the DDD conditions. Generalized mixed-effects regression models will be used for analyzing symptom and functioning outcomes. For treatment adherence, we will use mixed-effects Poisson and negative binomial regression models that are appropriate for count data [27]. Consistent with Aim 1, we will examine the main effect of year as well as the condition × year interaction to determine if the between-groups effects increase over time with annual optimization of the multivariate algorithm. Sensitivity analysis will be performed in the generalized linear mixed models to determine if there is significant variance associated with the clustering of students within coaches/clinicians.

As with the secondary analyses for Aim 1, we will explore whether treatment adherence mediates symptom outcomes using a linear mixed model with treatment adherence included as a time-varying covariate. Similarly, we will also examine whether this mediated effect is moderated by treatment tier. Another secondary analysis will focus on exploring the cost-effectiveness of the treatments. Specifically, an incremental cost-effectiveness ratio equal to the incremental service costs divided by incremental clinical outcomes for the SSD vs. DDD conditions will be computed. Health care service costs will consist of provider time spent delivering the intervention and cost of other service use (e.g., additional outpatient visits). Clinical health outcomes will consist of depressive and anxiety symptoms (e.g., the CAT-MH) and quality of life (e.g., Work and Social Adjustment Scale).

#### Statistical power

We plan to detect small effect size (*SD* = 0.26) in the SSD vs. DDD group difference at the end of the treatment [6]. We are assuming a linearly increasing effect size over time, modest correlation among the repeated assessments (*ρ* = 0.3), and a dropout rate of 5% between assessment periods. These assumptions are based on the preliminary analyses using the data from the pilot trial that took place in the same community college as the proposed study [11]. Power analysis in RMASS [77] with a Type I error rate of 5% for one-sided test and a power of 80% produced an estimated sample size of approximately *N* = 1000 (500 per group).

#### Missing data

The primary outcome analyses for Aim 1 on evaluating triage outcomes rely on the 8-week endpoint. Therefore, participants who discontinue assessments prior to 8 weeks will not be included in the analysis. For symptom severity and functioning outcomes, we plan to fit a linear mixed model that incorporates all available data for each participant, then estimate the 8-week endpoint difference between SSD and DDD from the complete set of available data. For adherence outcomes, given that the number of sessions through 8 weeks is fixed for a given treatment tier, we will use the planned number of sessions in the Poisson and negative binomial regression models. Regarding the outcome analyses for Aim 3 on evaluating adaptation outcomes, the proposed generalized linear mixed-effects regression models will account for the missing data. Specifically, the models assume that missing data, including dropouts, are missing at random (MAR; [27]. That is, a participant who drops out due to symptom deterioration or improvements would be MAR because the missing data are predictable from the observed outcomes prior to the dropout. We will also conduct a sensitivity analysis using a random effects pattern-mixture model, which is appropriate for data that are not missing at random (NMAR, [78]. If needed, we will use multiple imputation to impute values of missing predictors in the earlier data to use the full data in the updated prediction model [79, 80].

### Participant engagement and retention

To ensure adequate enrollment, we are implementing several recruitment strategies. First, we plan to recruit and enroll participants rapidly in the first few months of each academic year, which is the peak period for student enrollment in mental health services on college campuses. This includes campus-wide promotional emails, texts, banners, and flyers. We will also collaborate with the community college for the recruitment. This includes presenting the study in classrooms, having student ambassadors distribute flyers and answer questions at student events, and promotions on the community college’s learning information system/online course hub. To further increase enrollment, we will integrate the study as a service offering within the student health center at the community college, which would refer interested and potentially eligible students to STAND. Moreover, research staff will be available to answer any questions the participants may have during the enrollment and screening process.

A variety of strategies will be used to monitor and track participant completion of assessments and treatment sessions/online CBT sessions. Participants are provided with detailed instructions on the importance of their assessments for treatment personalization. Participants receive automatic assessment reminders via text or email. Late assessments and missed sessions will be tracked weekly by research assistants, who will reach out to participants manually by email or text. Additionally, we have provided monetary incentive to increase completion rates of assessments. Participants will be compensated up to $40 for completing the baseline assessments, $7 for each 8-week assessment (× 5 = up to $35 total), and $5 for each weekly assessment (× 40 = up to $200 total), for up to $275 in total possible incentives per participant. Lastly, we have plans to investigate the effectiveness of the strategies implemented for recruitment and engagement, which involves participant interviews. Participants will be asked to consent if they would like to be contacted for opportunities to participate. Findings from this smaller investigation will be used to inform engagement and retention strategies in the larger investigation.

### Data management, monitoring, and dissemination

Several measures will be taken to ensure the accuracy and completeness of the data during data collection, entry, and transmission. Monthly data management meetings will be conducted with the research team to troubleshoot any issues. Random data inspection will be conducted by the investigators and project coordinator. During the data analysis, investigators will work closely with the statistician to ensure all interpretations regarding the data are accurate and reliable. Interim analyses of the data will be conducted at 6 and 12 months annually. If the results show statistically overwhelming significant differences between groups (e.g., large effect sizes to the order of Cohen’s *d* > 0.8), or effect size differences much greater than expected, the study recruitment will be stopped.

A data safety monitoring board will monitor the execution of the study protocol and the safety of participants throughout the study. The board members consist of independent researchers at academic institutions with expertise in clinical trials. The board will be involved in reporting adverse events and conducting semi-annual reviews on participant safety. None of the cognitive and behavioral strategies in the three treatment tiers are experimental, nor are the medications that may be prescribed (e.g., SSRIs) for depression or anxiety in the highest level of care. No harmful effects have been identified with the multivariate or symptom severity based approaches to triaging and adapting level of care. Therefore, adverse effects of the treatment and of the triaging and adaptation methods are not expected, but symptoms will be closely monitored to reduce the possibility of adverse effects. Moreover, any adverse events will be continuously monitored throughout the study and any event will be followed to resolution or stabilization.

The current investigation uses the NIMH guidelines for assessing and reporting adverse events. The following adverse events are monitored: deaths, suicide attempts, study dropout, psychiatric hospitalizations, and clinical deterioration as defined as emergent suicidal ideation or suicidal plan, development of serious substance abuse, or the emergence of a new psychiatric or medical diagnosis or behavior posing a significant risk to the subject or others. The following rating system will be used to assess the seriousness of any adverse events: 0—no adverse event or within normal limits; 1—mild adverse event; 2—moderate adverse event; 3—severe adverse event resulting in hospitalization or a persistent or significant disability/incapacity; 4—life-threatening or disabling adverse event; 5—fatal adverse event. For events rated as 2–4, the principal investigators or another licensed psychologist will interview the participant and take appropriate action. All serious adverse events (i.e., events in the 3–5 range) will be reported immediately to the IRB and NIMH within 48 h, and all adverse events will be reported within 10 working days. Examples of serious adverse events include events that are life threatening or fatal, requires or prolongs hospitalization, or results in persistent or significant disability/incapacity. Within 1 week of a serious adverse event, the DSMB committee and the principal investigators will meet and discuss whether any modifications in the protocol are needed.

Under the principal investigators’ oversight, the research team will ensure that all data collection and entry methods are secure. All clinical and laboratory visits will be virtual. Informed consent will be obtained from participants before the baseline screening assessment. If needed, members of the study staff will meet with the prospective participants remotely via videoconferencing (and will ensure privacy at the beginning of the video call) to review the consent documents and provide an oral explanation of the study. Individuals will be given a chance to ask questions before making a considered decision about whether or not to participate in the study. Interview-based remote assessments will be conducted with privacy in mind, where both participants and assessors will be alone in private rooms with closed doors. All information entered through the web-based system will be encrypted with the secure sockets layer protocol. Access to all data on the server will be controlled by password protection. Different user profiles allow access to relevant sections of the database. Unique study identification numbers will be used to identify participants, and identifying information, including names, will not be transmitted via the web. The file linking names with the identification numbers (as well as the consent forms with names recorded on them) will be kept in a separate, secure file by the research coordinator.

## Discussion

The current study proposes to optimize personalization of mental health care using an innovative, multivariate stratification stepped care method in comparison with a traditional, symptom severity approach in mental health care triage and adaptation. Specifically, we will refine the multivariate predictive algorithm for triaging level of care and develop the novel predictive algorithm for the multivariate approach to adapting level of care for depression and anxiety. We will then compare this multivariate approach with the symptom severity approach with respect to treatment adherence, symptom reduction, and functional improvement.

### Innovation and impact

#### Predictive modeling for the community college population

Community college students are a high-risk group for depression and anxiety due to the experience of frequent psychosocial stressors [81]. However, depression and anxiety in this population is both understudied and undertreated. The current investigation targets the mental health needs of this at-risk population and is the first to focus on personalizing treatment in this population. Specifically, we will evaluate a predictive algorithm that uses an array of baseline information to estimate an individuals’ prognosis in treatment and stratify individuals into different tiers of treatment accordingly. We will then use the baseline features and dynamic features over time to adapt to the appropriate level of treatment. Information utilized in the algorithm include factors such as discrimination due to ethnic/cultural background, financial stress, sexual orientation, sleep problems, and gender identity, all of which has been linked with mental health status and care use in community college students [82]. Thus, the current investigation is the first to utilize a multivariate statistical tool for selecting and adapting level of care for community college students.

#### Theoretically informed variables for treatment personalization

Although multivariate models have been implemented in the clinical setting, the models have largely been limited to baseline evaluations and variables related to mental health status and functioning (e.g., [83–86]. Moreover, no studies to our knowledge have evaluated a comprehensive set of variables that are theoretically informed and suited to the needs of community populations. The current investigation includes factors that have been shown to drive mental health care needs, treatment engagement, and treatment response. This includes social determinants of mental health (e.g., discrimination, poverty), early life adversity and ongoing life stress (e.g., childhood trauma, financial stress), predisposing, enabling, and need influences on health service use (e.g., stigma, beliefs about treatment), and comprehensive mental health status (e.g., depressive symptoms, emotion regulation). These four theoretical clusters are overlapping and mutually reinforcing. For instance, discrimination is both a social determinant and a source of early adversity and ongoing life stress. Moreover, financial stress and poverty contribute to depression and anxiety, which in turn contribute to functional impairments and further downward mobility [87]. Therefore, we plan to test the variables from these four theoretical clusters as predictors of treatment response to inform the initial selection and later adaptation of level of care. Moreover, the models and variables will be iteratively reviewed and refined over the course of the study. That is, variables will be added and removed annually as appropriate to further improve predictive accuracy. Ultimately, the predictive models in the current investigation could be used as templates for community colleges regionally and state-wide. New community colleges can examine the predictive accuracy of our algorithm by collecting longitudinal data and determine if the predicted outcomes generalize to other community colleges, and further refine the algorithm as needed.

#### Advancement in personalized triaging to improve uptake and adherence

Dropout from intake to treatment is high across many clinical settings [88, 89]. Uptake in online therapy is also problematic, including in college student samples (e.g., [90, 91]. Relatedly, adherence and retention rates are low. Completion rates average at around 55% for digital therapies and 70% for clinician-delivered care [92, 93]. At college counseling settings, the modal number of appointments per student is only 1 per year, with an average number of less than 5 sessions attended per year [94]. Rates of uptake and engagement are particularly low for Latinx populations [95, 96], which is the largest group at the community college setting wherein the current investigation will take place. Greater personalization of triaging to level of care will likely improve both uptake and adherence. For instance, consideration of preference for online vs. clinician-delivered care alone may significantly improve these rates [97, 98]. We expect that the personalized approach—including adaptations to level of care and selection of digital and clinician-delivered treatment modules based on symptom presentations—will increase engagement and uptake in STAND.

#### Innovative measurement strategies for symptom severity

Current symptom severity will be measured using online computerized adapting testing (CAT-MH; [28–30]). The *CAT-MH* is a battery of adaptive mental health tests based on multidimensional item response theory. In clinical trials, the *CAT-MH* has been shown to increase measurement precision, reduce clinician burden, and minimize participant burden compared to traditional fixed length instruments [99–101]. This instrument is suitable for large-scale investigations as it reproduces information from large “banks” of symptom items (e.g., 389 items for depression and 431 items for anxiety). At the same time, the CAT-MH is substantially briefer than standard questionnaire batteries because of adaptive selection of a small set of items optimally targeted to a person’s level of severity at that point in time. It is also optimal for multiple assessments longitudinally because its adaptive nature minimizes responder bias that is associated with repeating the same item set.

#### Prevention and maintenance in mental health care

The current investigation utilizes the STAND model, which includes a self-guided online wellness program that specifically targets the prevention of depression and anxiety. Currently, most stepped care models do not include prevention, even though early intervention is likely lead to more positive outcomes and less patient distress and burden than intervening at the point of acute need [102]. Moreover, current clinical paradigms do not fully address the need for additional care during the maintenance phase even though maintaining gains is a cost-effective strategy for treating disorders such as depression [103]. Thus, the current investigation focuses not only on adapting level of care during the initial phase of treatment, but also using frequent assessments during the maintenance phase. These assessments will be used to inform monthly recommendations to either increasing level of care, staying in the current level of care, or re-initiating care. This approach allows for treatment adaptations to occur in a timelier manner, as opposed to waiting until non-responsiveness is observed at the end of the treatment.

### Limitations

Despite the areas of innovation and the methodological strengths of this personalized mental health care study, there are limitations worth noting. First, we focus on personalized mental health care in community colleges due to a greater need of accessible and effective psychological treatments in this population. That is, the variables for the level of care as well as the algorithm developed may be the most specific to the community college population. Additional studies are needed to evaluate the generalizability of the algorithms’ utility before the algorithm can be adapted to other settings. Second, treatment efficacy is a multi-faceted construct. We are focusing on treatment adherence, depressive and anxiety symptom level, and work/academic and social functioning because these aspects are the most relevant for community college students seeking treatment for depressive and anxiety disorders. It is noted that treatment efficacy may look different for other populations and disorders. For instance, treatment efficacy for developmental disorders may include factors such as meeting developmental milestones and parental and teacher ratings. These questions related to the application of the predictive algorithm to other disorders and settings warrant future investigation.

## Conclusion

The current investigation aims to advance personalized mental health care, particularly for diverse populations. Using a multivariate statistical approach, we aim to identify a sensitive set of predictors for the level of care needed and refine a predictive algorithm for mental health care triage and adaptation. We will be recruiting from a community college setting with diverse populations and greater need for accessible and effective mental health care. Additionally, we will explore treatment progress, symptom reduction, and functional improvement over time between the novel multivariate approach and the traditional symptom severity approach. At the completion of this study, we hope to produce a multivariate predictive algorithm that can be implemented in community college settings and be the foundation for the development and adaptation of personalized mental health care to other disorders and settings.

### Supplementary Information

**Additional file 1.** Appendix.

## Data Availability

The final trial dataset will be available in the NIMH Data Archive in accordance with the NIMH data sharing guidelines. Data will also be provided to other research groups upon request.

## Footnotes

1 Non-specialists without formal mental health credentials who are trained to provide or support low-intensity mental health services in community settings.
