# Supplementary material for: A randomized controlled trial on using predictive algorithm to adapt level of psychological care for community college students: STAND triaging and adapting to level of care study protocol
Source: Trials. 2023 Aug 9;24:508. doi: 10.1186/s13063-023-07441-7 (PMC10410881; doi:10.1186/s13063-023-07441-7)
Supplement: Supplementary file 1 — Additional file 1. Appendix. [file 13063_2023_7441_MOESM1_ESM.docx]

**Appendix**

**UNIVERSITY OF CALIFORNIA LOS ANGELES**

**CONSENT TO PARTICIPATE IN RESEARCH**

**STAND Finding and adapting the level of care study (Level of Care research study)**

**Thank you for signing up for the STAND at ELAC Level of Care research project!** This study is funded with a grant from the National Institute of Mental Health (NIMH) and is being conducted under the UCLA Depression Grand Challenge by Kate Taylor, PhD and Michelle Craske, PhD, from the Department of Psychiatry, at the University of California, Los Angeles (UCLA). You were selected as a possible participant in this study because you expressed interest in the STAND at ELAC program, you are eligible to receive services through the STAND at ELAC program, and you signed up for STAND at ELAC during the time period when we were recruiting participants for the Level of Care research study. Your participation in this research study is voluntary.

WHAT SHOULD I KNOW ABOUT A RESEARCH STUDY?

- Research studies are voluntary. Whether or not you take part is up to you.
- Someone will explain this research study to you.
- You can discuss this study with friends, family or your doctor.
- If you have any questions, you can ask the researchers for more information before deciding to participate.]
- You can agree to take part and later change your mind.
- Your decision will not be held against you.

WHAT is the study RESEARCHING?

The ***STAND Finding and Adapting the Level of Care*** (“Level of Care”) research study is investigating methods of finding the most appropriate level of care for each student and adapting care to maximize outcomes.

WHY IS THIS STUDY BEING DONE?

As you know from the STAND at ELAC general program consent, the STAND approach involves three levels of care and your level of care is determined based upon response to specific surveys regarding your symptoms. The researchers are interested in comparing two different methods of determining and adapting to the appropriate level of care for each individual within STAND. Findings from this study may enable us to develop and implement better ways of providing personalized care in the future.

WHAT IS THE DIFFERENCE BETWEEN PARTICIPATING IN THE REGULAR STAND AT ELAC PROGRAM AND THE STAND AT ELAC LEVEL OF CARE RESEARCH STUDY?

There are two primary differences for students participating in the regular STAND at ELAC program and the STAND at ELAC Level of Care research study. The first difference is that individuals in the research study will take more assessments/surveys regarding their symptoms and life experiences.

The second difference between the programs is how treatment is assigned. In the regular program, treatment is assigned based on presenting symptoms (we call this “solely symptom decision-making” or “SSD”). In the Level of Care research project, some people are assigned treatment based on SSD, while others are assigned treatment based on presenting symptoms in combination with other information that you provided to our program (we call this method, “data driven decision” or “DDD”). All participants regardless of group assignment will receive evidence-based treatments. The treatment is not experimental. Whichever group to which you are assigned (SSD or DDD) will remain the method by which future treatment decisions are made during your participation in the research study.

WHAT WILL I NEED TO DO IF I ENROLL IN THIS STUDY?

Over the 10-month research project duration, while receiving STAND at ELAC services, you will complete brief questionnaires sent every week and longer surveys sent every two months. We anticipate that your participation will be increased by 8 hours of time if you choose to participate in the Level of Care research study. More details about the different steps and types of data collected are provided below.

**Before you begin the study**: Following your agreement to participate in STAND at ELAC, if you decide to participate in the Level of Care research project, you will review and sign this consent form. You will be randomly assigned to either SSD or DDD group and then be routed to the initial assessments. Your group assignment will be confidential and will not be shared with you.

By agreeing to participate, you agree for any data that you have provided to STAND at ELAC prior to this consent (e.g., at account creation or eligibility screening) to be added to your STAND research and clinical record and used in accordance with the procedures outlined in this consent form.

**During the study:**

- **Baseline assessment** (about 1.5 hours)
  - Complete a series of online surveys and computer tasks.
- **Assignment to Treatment.** During the baseline assessment, you will be assigned to treatment by SSD or DDD as applicable and then be routed for the onboarding process relevant to your treatment assignment (e.g., wellness, digital program or clinical care).
- **Weekly & Other Research Assessments throughout the Study.**

Over the subsequent 40 weeks, the research team will ask you to complete approximately 8 hours of assessments including the following:

- - A brief survey once every week.
  - Other online surveys every eight weeks.

Completing these assessments is an important aspect of participating in the Level of Care research study. These data help us: monitor your progress through treatment, make informed decisions about your treatment, and evaluate and improve our service offering. As was described in the regular program consent, if you provide responses that indicate a need for social services or that you are potentially suicidal with a plan to harm yourself, a member of the extended STAND at ELAC team will reach out to you to check on your safety and connect you to support.

**Other Important Information about your Participation:**

- **Ongoing Communication:** During your participation, you will receive regular communication via e-mail, phone call or text from the study team.
- **Data Sharing:** By agreeing to participate, you also are agreeing to allow your data to be kept for use in future research to learn about, prevent or treat depression or other health-related problems.

HOW LONG WILL I BE IN THE STUDY?

If you enroll in the Level of Care research study, you will be expected to complete research assessments over a 40-week period. How often and for how long you engage with the resources available through your level of care will depend on you and your clinical/coaching team, if applicable. If at the end of the 40-week period, you are interested in continuing your participation in STAND at ELAC, you will have the option to continue with the regular program with access to services for several additional weeks (up to 52 weeks in total when combined with your research project participation).

HOW MANY PEOPLE WILL TAKE PART IN THIS STUDY?

We aim to recruit two hundred students per year (up to 1000 participants over 5 years) into the Level of Care research study.

IS THERE A CHANCE I WILL NOT BE ABLE TO PARTICIPATE IN THE RESEARCH PROJECT? IF SO, WHAT WILL HAPPEN?

The STAND at ELAC Level of Care research study has limited space available in each academic year. Therefore, if the treatment program selected for you based on your group membership has no space available, you will be able to continue to receive STAND at ELAC services outside of the research study. We may be able to place you on a waitlist. If you are found to not be eligible for regular STAND at ELAC or the Level of Care research project, our team, will provide you with information about other resources available to you and will help you navigate these to find an appropriate alternative for services.

WHAT KIND OF RISKS OR DISCOMFORTS COULD I EXPECT?

**Known risks and discomforts:** The possible risks and/or discomforts associated with the procedures described in this consent form include the following:

**Risks associated with mood and cognitive assessments:** The risks of answering questions about your mood, feelings, thinking or past experiences include fatigue, anxiety or discomfort. You can refuse to answer any question or end any research interview or treatment session, are welcome to discuss your emotions with the researchers or clinicians at any time; and can contact the principal investigators for additional emotional support if needed.

**Overall risks of loss of confidentiality:** One risk of participating in this study is that confidential information about you may be accidentally disclosed.  We will use our best efforts to keep the information about you secure.  Please see the Notice of Privacy Practices and regular STAND at ELAC consent form to understand our privacy and data security practices.

**Unknown risks and discomforts:** The services provided through this study may have side effects that no one knows about yet. The researchers will let you know if they learn anything that might make you change your mind about participating in the study.

ARE THERE ANY BENEFITS IF I PARTICIPATE?

**Possible benefits to you:** The possible benefits you may experience from being in this study might include improvement in depressive symptoms and earlier interventions. However, a response to treatments offered cannot be guaranteed in any participant and neither the degree of response nor the duration of response to one of the services offered can be reliably predicted at this time.

**Possible benefits to others in society:** This study aims to further medical knowledge and may improve future treatment of depression and anxiety.

WHAT OTHER CHOICES DO I HAVE IF I DON’T WANT TO OR AM UNABLE TO PARTICIPATE IN THE RESEARCH STUDY?

Your participation in the study is voluntary. If you decide not to take part in this study, or if you drop/withdraw from this study before it is completed, you may return to the regular STAND at ELAC program. If you are not eligible for the STAND at ELAC program and do not have or cannot afford a private psychologist or psychiatrist, we will recommend low cost treatment centers that may be able to help you.  Some specific resources available to you can be found through [this website](https://ucla.app.box.com/v/stand-elac-resource-guide).

CAN THE RESEARCHERS REMOVE ME FROM THIS STUDY?

The researchers may end your participation in this study for a number of reasons, such as if your safety and welfare are at risk, if you do not follow instructions, if you choose to receive treatment elsewhere (e.g., become a participant in another treatment study) or if you miss scheduled visits. For example, if you become ill during the research, you may have to drop out, even if you would like to continue. The investigators, Drs. Michelle Craske and Kate Taylor, will make the decision and let you know if it is not possible for you to continue. The decision may be made either to protect your health and safety, or because it is part of the research plan that people who develop certain conditions may not continue to participate. The researchers or the study sponsor might also decide to stop the study at any time. The team will provide you with information about available resources if this occurs.

Note that if you decide to stop being in the study, or are removed from the study, or the study is stopped, the data collected about you up to that point will remain part of the study and may not be removed from the study database.

HOW WILL MY INFORMATION BE KEPT CONFIDENTIAL?

Please refer to the regular STAND at ELAC program materials for details about how your information will be protected and shared for treatment payment and operations.

Information about you will be handled as confidentially as possible, but participating in research may involve a loss of privacy and the potential for a breach in confidentiality. Study data will be physically and electronically secured. As with any use of electronic means to store data, there is a risk of breach of data security. If you choose to participate in the research project, the following provisions also will apply:

**Use of Study ID to protect your identity:** As part of our commitment to your privacy, you have been assigned a unique study ID that will follow you throughout the course of the study. Study data containing your name or other information that could directly identify you is kept in secure, password-protected, and locked locations and will only be accessed by individuals who have been trained to protect your privacy.

We will replace your name with your Study ID for the records that relate to your assessments but we will maintain your name and contact information on records that are involving your treatment.

**What you can do to protect your privacy:** As part of this study you will be answering questions and completing tasks in this study that relate to your mood and mental health. Because this information can be sensitive, the study team will ask you to password protect your personal phone on which the apps will be used and to refrain from sharing your password with others.

**Retention of your data and records:** The researchers intend to keep the research data and records in a repository indefinitely.

**Who will have access to your data:** The team supporting the STAND at ELAC treatment program, the research study team members and other groups described below will have access to your information.

**Data sharing & broad consent**: By signing this consent form, you will give us permission to share your information beyond the treating providers and study team members.

If we do share information beyond the treating providers and researchers, the data that we will share will remain de-identified, meaning they will not contain identifying information.

Groups who may have access to de-identified information about you include:

- The study team members including investigators working at other research centers (academic or commercial).
- Other investigators not affiliated with the project who are investigating similar questions and who agree to protect the data. You will not be informed of the details of any specific research studies that might be conducted using your data, including the purposes of the research, and it is possible that you might not have chosen to consent to some of those specific research studies. Results from these studies may not be disclosed to you.
- Study monitors and auditors, for example from the NIMH or DMH, who make sure that the study is conducted properly.
- Readers and reviewers of scientific journals that may publish the results of this and other studies that involve your data but not your name or likeness.
- Data Archive repositories, for example The National Institutes of Health (NIH) and NIMH have developed a federation of data repositories called the NIMH Data Archive (NDA) to store the collection of de-identified data from participants in research studies related to mental health. The extensive information collected by these studies provides a rare and valuable scientific resource to the largest possible number of qualified investigators in an effort to achieve rapid scientific progress.

**Data sharing with you**: While there is some information regarding your symptom scores that we will share with you, we are unable to share most of the other assessment results from the study with you or your provider.

**Certificate of Confidentiality also protects you:** This research is covered by a Certificate of Confidentiality from the National Institutes of Health. This means that the researchers cannot release or use information, documents, or samples **that may identify you** in any action or suit unless you say it is okay. They also cannot provide them as evidence unless you have agreed. This protection includes federal, state, or local civil, criminal, administrative, legislative, or other proceedings. An example would be a court subpoena.

While there are these protections, the Certificate **will not stop** the type of reporting that federal, state or local laws require and that are outlined in the Notice of Privacy Practices. For example, it won’t stop mandated reporting of child or elder abuse or threats to harm yourself or others. The Certificate CANNOT BE USED to stop a sponsoring United States federal or state government agency from checking records or evaluating programs. The Certificate also DOES NOT prevent your information from being used for other research if allowed by federal regulations.

Researchers may release information about you when you say it is okay. For example, by participating in the STAND at ELAC program you have given permission to release certain information to insurers, medical providers or any other persons not connected with the research. The Certificate of Confidentiality does not stop you from willingly releasing information about your involvement in this research. It also does not prevent you from having access to your own information.

WILL I BE PAID FOR MY PARTICIPATION?

You will be compensated for completing research assessments in this study. Participants will receive up to $275, depending on completion of specific assessments. The compensation is provided as follows:

- $40 total for completed baseline assessments (of which $15 paid after program orientation; dependent on specific assessment completion)
- $5 per week for completed weekly survey paid each 8 weeks (up to $200 total)
- $7 for the additional surveys distributed each 8 weeks (x5) (up to $35 total).

Compensation will be provided as a Visa debit card offered through the Focus Blue Program by US Bank, or as an electronic gift card. The Visa debit card will be registered in your name and mailed to your home or designated address within 7-10 days upon completion of the baseline assessment. Once you have confirmed receipt of the card, it will be loaded with the amount earned after your assessment. Compensation earned by the end of every eight weeks will be loaded on this same Visa debit card. You have been provided with the schedule of fees associated with the card and will receive a printed copy when you receive the card in the mail or can ask to receive a copy from Study Staff.

ARE THERE ANY COSTS FOR TAKING PART IN THIS STUDY?

If you are receiving wellness or digital mental health services, no costs are anticipated for your participation. If you are receiving clinical care, just as there is with the regular STAND at ELAC program, you or your government insurance policy (if applicable) may be charged for certain services, such as medications or medical visits. The fees will be on a sliding scale and the clinician’s office will let you know in advance.

If you receive a prepaid Visa debit card to receive compensation for study participation, there is a chance you may incur fees for using the card in the following circumstances: 1) you use an out-of-network ATM, 2) you require more than 2 card replacements within a 12-month period, or 3) if your card carries a balance and you do not use the card for over 365 days.

WHO CAN I CONTACT IF I HAVE QUESTIONS ABOUT THIS STUDY?

**The Research Team:** You may contact the Principal Investigators, Michelle Craske at (310) 825-8403 or Dr. Kate Taylor at [kbtaylor@mednet.ucla.edu](mailto:kbtaylor@mednet.ucla.edu), (310) 267-5339, or contact our study team at [STANDelac@ucla.edu](mailto:STANDelac@ucla.edu) or (310) 872-4010 with any questions or concerns about the research or your participation in this study.

**LACDMH:** Clients served by the Los Angeles County Department of Mental Health directly-operated clinics or LE contractors with questions or concerns regarding the impact of their research activities on access to or quality of their usual care may contact the Los Angeles County Department of Mental Health Human Subjects Research Committee at [hsrc@dmh.lacounty.gov](mailto:hsrc@dmh.lacounty.gov).

**UCLA Office of the Human Research Protection Program (OHRPP):** If you have questions about your rights while taking part in this study, or you have concerns or suggestions and you want to talk to someone other than the researchers about the study, you may contact the UCLA OHRPP by phone: (310) 825-5344; by email: [mirb@research.ucla.edu](mailto:mirb@research.ucla.edu) or U.S. mail: UCLA OHRPP, 11000 Kinross Ave., Suite 211, Box 951694, Los Angeles, CA 90095-1694.

WHAT ARE MY RIGHTS IF I TAKE PART IN THIS STUDY?

**Rights of research subjects:** Taking part in this study is your choice. You can choose whether or not you want to participate. Whatever decision you make, there will be no penalty to you and you will not lose any of your regular benefits.

- You have a right to have all of your questions answered before deciding whether to take part.
- Your decision will not affect the medical care you receive from UCLA.
- If you decide to take part, you can leave the study at any time.
- If you decide to stop being in this study you should notify the research team right away. The researchers may ask you to complete some procedures in order to protect your safety.
- If you decide not to take part, you can still get medical care from UCLA.

HOW DO I INDICATE MY AGREEMENT TO PARTICIPATE?

**Consent to Audio/Video Recording and Observation:** By agreeing to participate, you agree to allow the observation in person, via audio or video recording of your interviews (if applicable), treatment and coaching sessions for the purposes of teaching and clinical supervision and quality assurance.

**Agreement to participate:** If you agree to participate in this study you should sign and date below. You will be asked to sign a separate form authorizing access, use, creation or disclosure of health information about you.

Signature

____________________

Date

**Agreement to be contacted for future studies**

Please also indicate below if you additionally agree to be contacted by UCLA researchers in the future to take part in other research studies, or to follow-up on your participation in this study.

Please check the appropriate box below and initial:

___ I agree to be contacted for future research studies

___ I do NOT agree to be contacted for future research studies
